# Knowledge, Attitude, and Behavior of the Pakistani Population Toward the Monkeypox Pandemic and the Associated Factors

**DOI:** 10.7759/cureus.73061

**Published:** 2024-11-05

**Authors:** Uzma Hafeez, Sara Bashir Kant, Syeda Sakina, Sohail Khan Raja, Amna Akbar, Muhammad Iftikhar Khattak, Mumtaz Ahmed, Sarosh Khan Jadoon, Sabahat Tasneem

**Affiliations:** 1 Public Health/Community Medicine, Azad Jammu Kashmir Medical College, Muzaffarabad, PAK; 2 Dermatology, Dr. Sakina Clinics, Wah Cantt, PAK; 3 Pulmonology, Azad Jammu Kashmir Medical College, Muzaffarabad, PAK; 4 Emergency and Accident, District Headquarter Hospital, Jhelum Valley, Muzaffarabad, PAK; 5 Research and Development, Celestial &amp; Dimanche, Muzaffarabad, PAK; 6 Pathology, Azad Jammu Kashmir Medical College, Muzaffarabad, PAK; 7 General Surgery, Combined Military Hospital, Muzaffarabad, PAK; 8 Public Health, Health Services Academy, Islamabad, PAK

**Keywords:** attitude, behavior, knowledge, monkeypox, sociodemographic factors

## Abstract

Background

Monkeypox (Mpox) is a virulent disease caused by orthopoxvirus. Mpox is emerging as a major global health threat. Currently, more than 100 countries are facing outbreaks. Pakistan, too, is witnessing the spread of this virus, with 11 confirmed cases and one death since its first detection in April 2023. Mpox infection can be diagnosed using polymerase chain reaction (PCR) and treated with antiviral agents. The smallpox vaccine is also proven to be effective against Mpox.

Methodology

This cross-sectional survey aimed to evaluate the knowledge, attitude, and behaviors (KAB) of the Pakistani population toward the Mpox pandemic and determine the factors affecting it. Data were collected through Google Forms using a validated questionnaire to assess the population’s KAB. In total, 1,511 individuals were included in the final analysis.

Results

Study participants had good knowledge of the disease, poor attitude toward Mpox risk and severity, and poor behavior with low adherence to recommended protocols. Overall, 58% (n = 888) of the participants were male, and most of the respondents were aged between 18 and 30 years (n = 743, 49.2%). Most participants were married (n = 983, 65.1%), from urban areas (n = 837, 55.4%), and living in shared households (n = 876, 58%). Age showed a significant relationship with knowledge level and behavior, but not with attitude. The 18-30-year age group demonstrated higher knowledge levels (p = 0.007), regardless of gender. Shared households were significantly associated with a higher incidence of good knowledge (p < 0.05) compared to independent households (p = 0.038). Additionally, higher income was linked to better attitudes and behaviors. KAB outcomes also varied significantly based on marital status, individual education level, and parents’ education levels.

Conclusions

Population dynamics such as cultural norms, religious beliefs, misperceptions about the disease associated with sexual behavior, health literacy, education level, rural and urban division of the population, gender role, migrant and refugee population, poverty, cost-seeking healthcare, and distrust in the government and healthcare system should be considered when constructing a public health policy because the behavior of the population is important for the implementation of preventive measures.

## Introduction

Monkeypox (Mpox) is caused by an infection with the Mpox virus (orthopoxvirus), a member of the Poxviridae family of viruses [1]. Symptoms of Mpox resemble those of smallpox but are milder and not typically deadly. First discovered in 1958 in apes held for research, Mpox was ignored by Western researchers. The first case of human Mpox was identified in 1970 in the Democratic Republic of Congo. The first Mpox outbreak in the United States was reported in 2003 [2]. Clades I and II are two types of Mpox. The fatality rate of Clade I is approximately 10%, whereas that of Clade II is 6% [3,4]. Mpox presents with mild symptoms such as fever, rash, and lymph node swelling [5]. It may lead to common complications, including bacterial superinfection, pigmentation, corneal scarring, pneumonia, dehydration, sepsis, and encephalitis [6].

Currently, more than 100 countries are facing Mpox outbreaks [7]. An international survey of Mpox in 16 countries showed that 98% of infected people were bisexual or gay and 41% had acquired immunodeficiency syndrome, with a median age of 38 years. Overall, 95% of viral transmissions occur through sexual contact. Most participants had mild symptoms, with only 13% requiring hospital admission [8]. Risk Communication & Community Engagement plans focus on spreading awareness among the public so that they can protect themselves and prevent the spread of the virus. The main objectives are intense, clear, and targeted interventions that do not cause stigma in high-risk populations [9]. Mpox cases have risen dramatically in 10 central and western African countries over the last 20 years. Several outbreaks have been reported in non-endemic countries since May 2022. The World Health Organization (WHO) has declared Mpox outbreaks a Public Health Emergency of International Concern (PHEIC) [10,11]. Few studies have been conducted on public preventive behaviors against viruses [12].

As a result of the discovery of the first two cases of the Mpox virus in Pakistan, health ministry authorities have taken preventative measures to limit the dissemination of the disease by placing airports and medical facilities on high alert. Pakistan has experienced two cases of Mpox, both of which have been successfully treated. The initial case was discovered when a resident of Islamabad, who had just returned from an overseas flight, tested positive for the virus. He was deported from Saudi Arabia and exhibited symptoms of Mpox. The patient displayed viral illness symptoms upon arriving in Pakistan on April 17, 2023, with the first Mpox case confirmed by the National Institute of Health. He was isolated at the Pakistan Institute of Medical Sciences, while another passenger with similar symptoms was quarantined at home. Both individuals were stable. Health authorities investigated contacts, set up a control room, and held virtual meetings with relevant departments [13]. Despite no indication of local Mpox transmission, authorities placed airports on high alert for medical screening. Public health officials increased surveillance through lab diagnostics, contact tracing, and rapid identification of suspected cases to provide care and prevent further spread [14]. Until now, 11 confirmed cases and one death have been reported from Pakistan [15].

Mpox outbreaks have attracted less research attention than COVID-19 and people are not well aware of Mpox and its threats [16]. Misinformation about Mpox generates stigmas and incorrect beliefs [17]. People are unwilling to receive the Mpox vaccination. They opt for the “no vaccine” option if it is not mandatory to vaccinate [18]. Therefore, we believe that there is a knowledge gap regarding understanding disease transmission, prevention, and treatment; lack of general awareness about symptoms, risks, and negative public health impact; and incorrect beliefs such as impotence, which can hinder public understanding and response to the disease and reluctance to receive the Mpox vaccine leading to a negative attitude toward taking preventive steps to reduce the exposure risk to the Mpox virus. The only way to fill this knowledge gap is to identify the predictors of people’s behavior toward the outbreak and design public health interventions tailored to people’s needs. To design clear, appropriate, and uniform communication, we need to research and delve deeper into the social and demographic factors related to people’s behaviors. Few studies have been conducted in Pakistan to determine the population’s knowledge and behavior toward the Mpox virus.

This study aimed to determine the knowledge, attitude, and behaviors (KAB) of the Pakistani population and the associated factors regarding Mpox.

## Materials and methods

This study was evaluated and accepted by the Ethical Review Board of Abbas Institute of Medical Sciences (AIMS), Muzaffarabad, Azad Kashmir (approval number: AIMS/1018/2022).

Study design and inclusion and exclusion criteria

This cross-sectional study was conducted in 12 different cities of Pakistan, including Islamabad, Rawalpindi, Peshawar, Lahore, Karachi, Multan, Bahawalpur, Hyderabad, Quetta, Muzaffarabad, Rawalakot, and Mirpur between December 2022 and July 15, 2023. The participants were selected using non-probability convenience sampling to gather initial insights due to time and budget constraints, limited manpower, ease of participant access, and the rarity of the disease, which made random or systematic sampling challenging. The included participants were from Pakistan, aged above 18 years, and were able to read English. Individuals under 18 years of age who could not understand English and foreigners were excluded to ensure that participants fully comprehended the study materials, enhancing data accuracy, reliability, and validity. This also created a homogeneous population, reducing confounding variables and making the findings more relevant to the target group.

Sample size and data collection

The sample size was determined using Cochran’s formula: n = (z^2^.pq)/e^2 ^= 385, where q = 1-p, z = 1.96 (at a 95% confidence level), e = 0.05, and the probability (p) was assumed to be 50% when there was no idea of the probability of the event to occur [19]. In total, 385 was the minimum required sample size. Using non-probability convenience sampling, we collected responses from 1,511 individuals to improve the precision of estimates and reduce the margin of error, making the results more reliable.

Questionnaire structure and measures

The questionnaire was adapted from a previous study in Pakistan [20]. The questionnaire was modified for our study, as the previous study included participants from the medical field and we included the general population. The questionnaire was written in the English language. The questionnaire contained four parts. The first part included sociodemographic information, and the second part contained questions about knowledge, with each item’s variable value marked as 1 (yes) and 0 for (no). The third part included questions about attitudes, with values ranging from 1 (strongly disagree) to 5 (strongly agree). The last part included questions on behaviors (seven items), with values ranging from 1 (never) to 5 (always). Cronbach’s alpha was used to determine the validity of the questionnaire [21]. The first 50 responses were considered for the test and excluded from the main study. Cronbach’s alpha values for knowledge, attitude, and behavior were 0.831, 0.734, and 0.751, respectively.

Data collection and analysis

Participants provided written consent before they participated in the study via a Google Forms link with the questionnaire. Data were collected by sending Google Forms links through all social media platforms, such as WhatsApp and Facebook, which are widely used in Pakistan. The authors involved in this study contacted friends and family members in different areas of Pakistan and requested them to spread the questionnaire through social media in their own areas. There were no incentives or rewards for participation. The participants were requested to complete the forms of their free will. Text messages and voice notes were sent with a Google Forms link to briefly explain the context and purpose of the study. The data were then transferred to SPSS Statistics version 25.0 (IBM Corp., Armonk, NY, USA), and missing values were replaced with adjacent column values. Frequencies and associations were determined using statistical analyses. The question items in the KAB parts were computed and summed on SPSS by the addition of item values using the “transform” and “compute variable” functions in SPSS. The sums of the question items were recoded as “KnowledgeSum,” “AttitudeSum,” and “BehaviorsSum,” using the transform function (the sum was recoded into different variables) in SPSS and divided into two categories for regression analysis. The median value was used as the cutoff point. The medians for knowledge, attitude, and behavior were 5, 22, and 20, respectively. The two categories based on the median for knowledge were “less than 5,” which was labeled as poor and “equal or more than 5,” which was labeled as good value for knowledge. The same rule was applied to attitudes and behaviors. Through cross-tabulation, a chi-square test was performed using SPSS to determine the relationship between these variables and KAB. The significance of the association was determined using p-values <0.05). The ordinal logistic regression analysis was used to confirm the association of demographic variables with KAB.

## Results

General characteristics of the study participants

**Figure 1:**
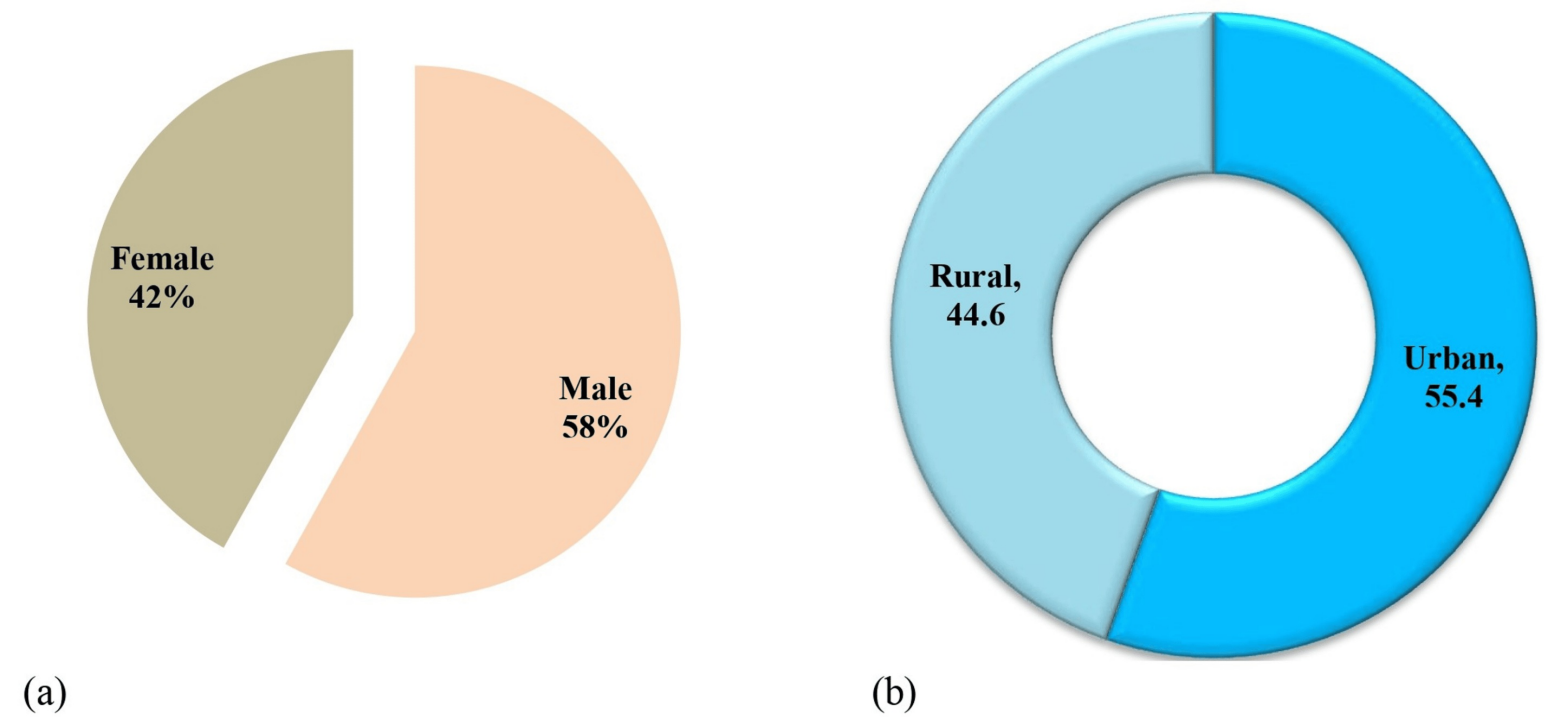
Graphical representation of participants’ demographic characteristics. (a) Gender. (b) Residence.

**Figure 2:**
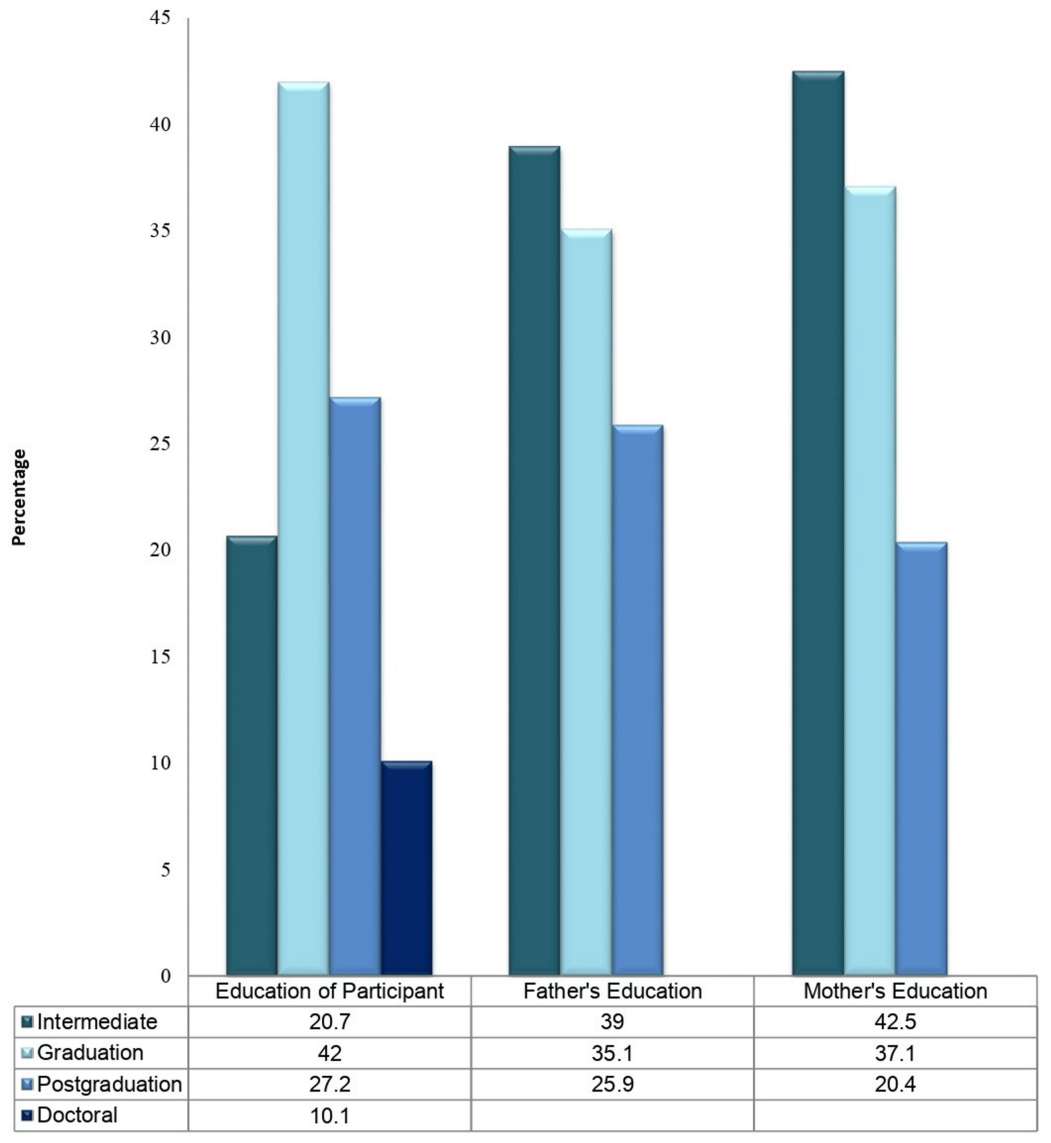
Education levels of fathers and mothers.

Table 1 presents the general characteristics of the participants. Data were collected from 1,511 individuals. Overall, 58% of the participants were male and 42% were female (Figure 1). Most respondents were aged between 18 and 30 years (49.2%). Most participants were married (65.1%), from urban areas (55.4%), and living in shared households (58%). Further, 42% of the individuals were university graduates, and 26.3% had no family members who worked in the medical field. Nearly one-third of the participants (34%) had a monthly income of more than 100,000 per year. Regarding fathers’ education, 25.9% of the participants were postgraduates, while 39% had an intermediate level of education. Only 20.4% of the participants’ mothers had a postgraduate level of education, whereas 42.6% had an intermediate level of education (Table 1, Figures 1, 2).

**Table 1:** Descriptive statistics: demographic variables. The p-values were obtained by the chi-square test in cross-tabs.

Variables	Categories	Frequency (percentage)	P-value		
			Knowledge	Attitude	Practice
Gender	Male	888 (58.1)	0.794	0.382	0.151
	Female	623 (41.2)			
Age (years)	18–30	743 (49.2)	0.007	0.459	0.002
	31–45	602 (39.8)			
	Above 45	166 (11.0)			
Legal marital status	Single	354 (23.4)	0.520	0.007	0.026
	Married	983 (65.1)			
	Divorced/Widowed	174 (11.5)			
Residence	Urban	837 (55.4)	0.019	0.249	0.014
	Rural	674 (44.6)			
Household	Live alone/Independent	635 (42.0)	0.038	0.912	0.208
	Shared household	876 (58.0)			
Education	Inter (12 years)	313 (20.7)	0.004	0.108	0.000
	Graduation	634 (42.0)			
	Masters	411 (27.2)			
	Doctoral (PhD)/postdoctoral	153 (10.1)			
Father’s education	Intermediate or less	590 (39.0)	0.000	0.758	0.007
	Graduation	530 (35.1)			
	Postgraduation	391 (25.9)			
Mother’s education	Intermediate or less	643 (42.6)	0.001	0.048	0.000
	Graduation	560 (37.1)			
	Postgraduation	308 (20.4)			
Family income	<50,000 PKR	431 (28.5)	0.003	0.512	0.004
	50,000–100,000 PKR	565 (37.4)			
	>100,000 PKR	515 (34.1)			
Family members in the medical field	Father	323 (21.4)	0.000	0.000	0.000
	Mother	281 (18.6)			
	Spouse	330 (21.8)			
	Children	179 (11.8)			
	None	398 (26.3)			

Table 1 shows that age had a significant relationship with knowledge level and behavior, but not with attitude. There was a significant relationship between younger age (18-30 years) and having higher knowledge levels (p = 0.007). However, there were no significant differences between males and females regarding their attitudes or knowledge levels. Shared households were associated with a significantly higher incidence of good knowledge than were independent households (p < 0.05). In addition, a high income was associated with a higher incidence of good attitudes and behaviors. However, there were significant differences in KAB outcomes according to the marital status of the residents, the education level of the individual, the father’s education level, and the mother’s education level.

Tables 2-4 show the participants’ KAB levels. Table 3 indicates a generally low level of agreement with proactive attitudes toward Mpox. The overall lack of strong endorsement for preventive actions suggests a disconnect between knowledge and attitudes. The ordinal regression analysis confirmed the association of demographic variables with KAB. The gap in knowledge, particularly regarding vaccination accessibility, may contribute to the observed behavioral patterns in Table 4. It further highlights this disconnect in behavior, revealing a critical gap in adherence to recommended preventive behaviors, which is concerning given the participants’ reported knowledge of Mpox (Tables 2-4).

**Table 2:** Survey descriptive analysis: knowledge. N: frequency; %: percentage

Knowledge	Yes		No	
Variable	N	%	N	%
Do you have knowledge of monkeypox?	1,107	73.3	404	26.7
Do you know how monkeypox is transmitted from one individual to another?	1,132	74.9	379	25.1
Do you know that monkeypox can cause pandemics and death?	1,113	73.7	398	26.3
Do you know how monkeypox spreads from endemic to non-endemic areas?	1,148	76	363	24
Do you know ways to prevent the spread of monkeypox?	1,005	66.5	506	33.5
Do you know about the tests through which the monkeypox is diagnosed?	884	58.5	627	41.5
Do you know about the vaccination and treatment of monkeypox?	824	54.5	687	45.5
Do you know the nearest place where you can receive treatment and a vaccine for monkeypox?	740	49	771	51

**Table 3:** Survey descriptive analysis: attitude. SD: strongly disagree; D: disagree; N: neutral; A: agree; SA: strongly agree; N (%): frequency (percentage)

Attitude	SD	D	N	A	SA
	N (%)	N (%)	N (%)	N (%)	N (%)
Do you believe that you are at risk of monkeypox?	44 (2.9)	720 (47.7)	13 (0.9)	462 (30.6)	272 (18)
Do you believe that monkeypox can cause pandemics or death?	50 (3.3)	653 (43.20	22 (1.5)	536 (35.5)	250 (16.5)
Do you think there should be a lockdown in areas reporting cases of monkeypox?	53 (3.5)	682 (45.1)	31 (2.1)	506 (33.5)	239 (15.8)
Do you believe that traveling from endemic areas (Africa, Nigeria, etc.) to non-endemic areas must be banned?	33 (2.2)	652 (43.2)	53 (3.5)	506 (33.5)	267 (17.7)
Do you believe that there is an increased risk of monkeypox during social gatherings and events?	133 (8.8)	556 (36.8)	24 (1.6)	548 (36.3)	250 (16.5)
Do you believe that the smallpox vaccine is effective against monkeypox?	40 (2.6)	766 (50.7)	35 (2.3)	457 (30.2)	213 (14.1)
Do you believe that there is a need for more awareness among the general public about monkeypox?	110 (7.3)	582 (38.5)	27 (1.8)	440 (29.1)	352 (23.3)

**Table 4:** Survey descriptive analysis: behavior. N (%): frequency (percentage)

Behavior	Never	Rarely	Sometimes	Often	Always
	N (%)	N (%)	N (%)	N (%)	N (%)
If you are infected with monkeypox, will you quarantine yourself?	359 (23.8)	367 (24.3)	285 (18.9)	287 (19)	213 (14.1)
Will you follow restrictions when there is a monkeypox pandemic?	381 (25.2)	253 (16.7)	380 (25.1)	342 (22.6)	155 (10.3)
To prevent monkeypox, do you avoid crowds and gatherings?	257 (17)	370 (21)	457 (30.2)	317 (21)	163 (10.8)
Do you wash your hands frequently out of fear of monkeypox?	301 (19.9)	368 (24.4)	398 (26.3)	290 (19.2)	154 (10.2)
Did you receive vaccination against smallpox or monkeypox?	442 (29.3)	281 (18.6)	329 (21.8)	295 (19.5)	164 (10.9)
Did you obtain information about monkeypox on television?	248 (16.4)	303 (20.1)	458 (30.3)	302 (20)	200 (13.2)
Did you get information about monkeypox on the Internet?	365 (24.2)	279 (18.5)	438 (29)	285 (18.9)	144 (9.5)

**Figure 3:**
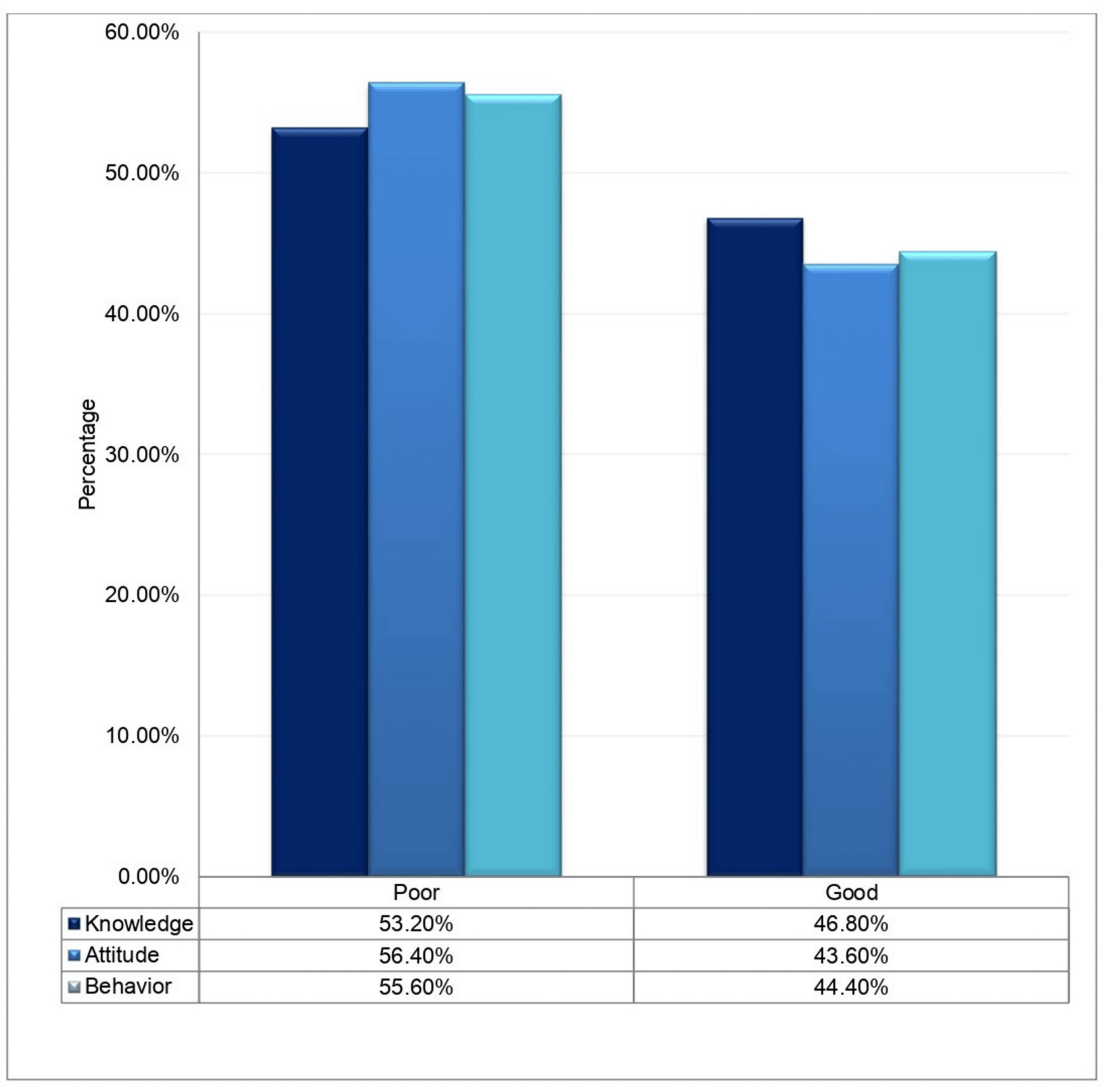
Percentages of good and poor knowledge, attitude, and behavior.

Figure 3 shows the percentages of the outcome variables.

The ordinal logistic regression analysis confirmed the association of demographic variables with KAB. Although participants exhibited a foundational level of knowledge about Mpox, this awareness did not translate into strong attitudes or effective behaviors aimed at prevention and control (Tables 5-7).

**Table 5:** Ordinal logistic regression analysis: knowledge. Sig.: p-value obtained by the chi-square test. aOR: the odds ratio obtained after the variable was adjusted for “Education” as a co-variable. OR: odds ratio; CI: confidence interval; aOR: adjusted odds ratio

Variables	Categories	OR	CI (95%)	Sig.	aOR	CI (95%)	Sig.
Gender	Male	0.973	0.792–1.195	0.794	0.969	0.789–1.190	0.763
	Female	_	_	_	_	_	_
Age (years)	18–30	1.194	0.962–1.482	0.107	0.585	0.416–0.824	0.002
	31–45	1.688	1.202–2.371	0.002	0.706	0.500–0.908	0.049
	Above 45	_	_	_	_	_	_
Legal marital status	Single	0.895	0.702–1.142	0.372	1.225	0.851–1.764	0.275
	Married	0.821	0.570–1.182	0.290	1.090	0.788–1.507	0.603
	Divorced/Widowed	_	_	_	_	_	_
Residence	Urban	1.276	1.041–1.564	0.019	0.786	0.641–0.964	0.021
	Rural	_	_	_	_	_	_
Household	Live alone/Independent	0.805	0.656–0.988	0.380	1.240	1.010–1.522	0.040
	Shared household	_	_	_	_	_	_
Education	Intermediate (12 years)	0.678	0.516–0.890	0.005	0.936	0.635–1.381	0.739
	Graduation	0.692	0.515–0.930	0.015	0.634	0.444–0.904	0.012
	Masters	1.071	0.726–1.579	0.730	0.647	0.446–0.904	0.022
	Doctoral/Postdoctoral	_	_	_	_	_	_
Father’s education	Intermediate or less	1.633	1.289–2.069	0.000	1.020	0.783–1.328	0.883
	Graduation	0.969	0.748–1.255	0.812	1.675	1.286–2.183	0.000
	Postgraduation	_	_	_	_	_	_
Mother’s education	Intermediate or less	1.542	1.227–1.938	0.000	0.705	0.536–0.926	0.012
	Graduation	1.417	1.078–1.862	0.012	1.085	0.821–1.433	0.567
	Postgraduation	_	_	_	_	_	_
Monthly family income	<50,000 PKR	1.001	0.779–1.286	0.993	1.459	1.123–1.895	0.005
	50,000–100,000 PKR	0.692	0.534–0.895	0.005	1.452	1.138–1.851	0.003
	>100,000 PKR	_	_	_	_	_	_
Family members in the medical field	Father	1.407	1.020–1.942	0.038	1.944	1.438–2.629	0.000
	Mother	1.056	0.777–1.436	0.726	2.718	1.983–3.727	0.000
	Spouse	0.861	0.597–1.242	0.424	2.044	1.515–2.758	0.000
	Children	0.517	0.383–0.699	0.000	1.665	1.161–2.386	0.006
	None	_	_	_	_	_	_

**Table 6:** Ordinal logistic regression analysis: attitude. Sig.: p-value obtained by the chi-square test. aOR: the odds ratio obtained after the variable was adjusted for “Education” as a co-variable. OR: odds ratio; CI: confidence interval; aOR: adjusted odds ratio

Variables	Categories	OR	CI (95%)	Sig.	aOR	CI (95%)	Sig.
Gender	Male	1.096	0.892–0.348	0.382	0.895	0.734–1.111	0.335
	Female	_	_	_	_	_	_
Age (years)	18–30	0.884	0.712–1.098	0.266	1.126	0.799–1.586	0.498
	31–45	0.862	0.613–1.212	0.392	1.023	0.722–1.451	0.896
	Above 45	_	_	_	_	_	_
Legal marital status	Single	0.725	0.569–0.926	0.010	1.711	1.180–2482	0.005
	Married	0.590	0.407–0.855	0.005	1.230	0.882–1.715	0.223
	Divorced/Widowed	_	_	_	_	_	_
Residence	Urban	1.128	0.919–1.384	0.249	0.895	0.730–1.099	0.291
	Rural	_	_	_	_	_	_
Household	Live alone/Independent	0.988	0.804–1.1214	0.912	1.010	0.822–1.242	0.923
	Shared household	_	_	_	_	_	_
Education	Intermediate (12years)	0.756	0.576–0.993	0.044	1.420	0.960–2.101	0.079
	Graduation	0.714	0.531–0.961	0.260	1.069	0.747–1.530	0.715
	Masters	0.709	0.480–1.048	0.085	1.011	0.693–1.475	0.954
	Doctoral (PhD)/Postdoctoral	_	_	_	_	_	_
Father’s education	Intermediate or less	1.086	0.857–1.375	0.495	0.945	0.726–1.230	0.674
	Graduation	1.003	0.775–1.299	0.979	1.058	0.812–1.378	0.678
	Postgraduation	_	_	_	_	_	_
Mother’s education	Intermediate or less	0.985	0.783–1.240	0.899	0.717	0.545–0.943	0.017
	Graduation	1.373	1.045–1.803	0.023	0.707	0.534–0.936	0.015
	Postgraduation	_	_	_	_	_	_
Monthly family income	<50,000 PKR	0.882	0.685–1.135	0.328	0.105	0.850–1.435	0.456
	50,000-100,000 PKR	0.871	0.673–1.127	0.292	0.983	0.770–1.254	0.888
	>100,000 PKR	_	_	_	_	_	_
Family members in the medical field	Father	1.511	1.093–2.087	0.012	0.719	0.534–0.969	0.030
	Mother	1.302	0.954–1.777	0.096	1.078	0.793–1.464	0.632
	Spouse	0.667	0.452–0.984	0.041	0.948	0.707–1.270	0.719
	Children	1.382	1.026–1.861	0.330	0.479	0.329–0.697	0.000
	None	_	_	_	_	_	_

**Table 7:** Ordinal regression analysis: behavior. Sig.: p-value obtained by the chi-square test. aOR: the odds ratio obtained after the variable was adjusted for “Education” as a co-variable. OR: odds ratio; CI: confidence interval; aOR: adjusted odds ratio

Variables	Categories	OR	CI (95%)	Sig.	aOR	CI (95%)	Sig.
Gender	Male	0.859	0.699–1.057	0.151	1.160	0.943–1.427	0.160
	Female	_	_	_	_	_	_
Age (years)	18–30	1.441	1.160–1.791	0.001	0.656	0.466–0.922	0.015
	31–45	1.439	1.026–2.018	0.035	0.997	0.706–1.408	0.988
	Above 45	_	_	_	_	_	_
Legal marital status	Single	0.768	0.602–0.980	0.034	1.594	1.100–2.309	0.014
	Married	0.626	0.433–0.906	0.013	1.226	0.880–1.706	0.228
	Divorced/Widowed	_	_	_	_	_	_
Residence	Urban	1.293	1.054–1.586	0.014	0.777	0.633–0.954	0.016
	Rural	_	_	_	_	_	_
Household	Live alone/Independent	0.876	0.714–1.076	0.208	1.145	0.932–1.407	0.196
	Shared household	_	_	_	_	_	_
Education	Intermediate (12 years)	0.496	0.377–0.653	0.000	1.715	1.159–2.535	0.007
	Graduation	0.635	0.472–0.853	0.003	0.855	0.598–1.224	0.393
	Masters	0.578	0.391–0.855	0.006	1.092	0.751–1.590	0.644
	Doctoral (PhD)/Postdoctoral	_	_	_	_	_	_
Father’s education	Intermediate or less	1.437	1.1355–1.821	0.003	0.869	0.667–1.133	0.299
	Graduation	1.068	0.824–1.384	0.617	1.309	1.005–1.706	0.046
	Postgraduation	_	_	_	_	_	_
Mother’s education	Intermediate or less	1.927	1.528–2.429	0.000	0.510	0.387–0.673	0.000
	Graduation	1.905	1.446–2.511	0.000	0.995	0.752–1.315	0.970
	Postgraduation	_	_	_	_	_	_
Monthly family income	<50,000 PKR	1.341	1.042–1.726	0.023	1.042	0.800–1.356	0.763
	50,000–100,000 PKR	0.911	0.702–1.181	0.481	1.426	1.118–1.819	0.004
	>100,000 PKR	_	_	_	_	_	_
Family members in the medical field	Father	1.832	1.326–2.532	0.000	1.533	1.131–2.078	0.006
	Mother	1.307	0.960–1.779	0.089	2.823	2.056–3.874	0.000
	Spouse	0.859	0.593–1.246	0.424	2.055	1.521–2.777	0.000
	Children	0.641	0.473–0.869	0.004	1.333	0.924–1.921	0.124
	None	_	_	_	_	_	_

## Discussion

Participants demonstrated a good level of knowledge regarding Mpox and its prevention and testing, though there were gaps in awareness about vaccination locations and procedures. The attitude of participants toward the risk and severity of Mpox was generally poor, with low recognition of the severity of the disease and limited support for preventive measures such as lockdowns. Participants showed poor behavior in adhering to preventive measures, with low rates of quarantine, following restrictions, hand hygiene, and vaccination uptake.

Among the 58% of males surveyed, 73.7% demonstrated good knowledge of Mpox, 66.5% were aware of its prevention, 58.5% knew about available tests, and 54.5% had information about vaccination. However, only 49% knew the location of the nearest vaccination facility. This shows a higher level of knowledge about risk and interest in disease prevention. After the COVID-19 pandemic, people became aware of the risks associated with pandemics. Knowledge regarding how to diagnose Mpox was limited to 58% of the participants. People may not report to medical facilities or obtain a confirmed diagnosis of Mpox because they are unaware of the procedure. Participants displayed a generally low perception of the risk and severity of Mpox (52.4%). Only 18% of participants believed that there was a risk of contracting Mpox, and only 16.5% considered it a potentially fatal disease. Additionally, 17.7% were willing to travel despite the risk, while 14.1% believed that the smallpox vaccine is effective against Mpox. Only 15.8% strongly supported lockdown measures, and 23.3% strongly favored a mass awareness campaign about the disease. The stronger agreement for a mass awareness campaign indicates recognition of the need for better education concerning the disease and its prevention (Table 2).

The participants’ behavior toward Mpox reflects a low level of adherence to preventive measures, such as quarantine, following restrictions, and receiving the vaccine. Only 14.1% of participants indicated they would quarantine if infected with Mpox, 10.3% said they would follow restrictions, while 10.8% would avoid them. Additionally, 10.2% stated they would wash their hands frequently, and just 10.9% agreed to receive the vaccine (Table 3).

In a survey of the US public, 47% of the respondents marked their knowledge of the Mpox virus as either poor or very poor. Healthcare workers are important sources of information. The willingness to take the Mpox vaccine was linked to having received the COVID-19 vaccination (odds ratio (OR) = 32.1, 95% confidence interval (CI) = 16.7-61.7) [20]. There is a gap in the knowledge regarding Mpox among clinicians. Therefore, they cannot effectively participate in the diagnosis, management, or prevention of the disease effectively [22]. In a KAB survey of 179 clinicians, their knowledge of Mpox was reported to be unsatisfactory. The surveyed clinicians showed little interest in following precautions, and only 40% of them were vaccinated against smallpox [23]. A Jordanian survey studied the level of knowledge about Mpox, the factors associated with behaviors toward the MPOX virus, and conspiracy beliefs among students. They found that only 161 of 615 students knew that the smallpox vaccine was available for Mpox. Additionally, they found that lower levels of knowledge, being female, and affiliation with non-medical health institutions were significantly associated with higher conspiracy beliefs [24]. In a cross-sectional study of 558 Emirati students, 20% had poor, 57% had moderate, and 23% had good knowledge of Mpox according to Bloom’s cutoff point. Older age, female gender, participation in medical courses, learning about human Mpox while in school, and a history of chickenpox infection were positively associated with better knowledge [25]. In this cross-sectional survey, 46.8% had good knowledge, 43.6% had a good attitude, and 44.4% had good behavior about Mpox.

In a KAB study among Pakistani students, Kumar et al. reported that among 946 respondents, 76.7% had average knowledge, with very few individuals having good knowledge (6.3%). They found that the degree of knowledge was significantly associated with the type of academic degree, discipline, and region. Regarding attitudes, they reported that the attitudes of most participants were neutral. They highlighted the necessity of teaching students about the Mpox virus to increase public awareness, which would increase compliance with precautionary instructions [26]. In our study, family members in the medical field and marital status had positive impacts on attitudes.

Education affects preventive attitudes. People with higher education can better understand the need for prevention; therefore, they have a positive attitude toward prevention. A cross-sectional survey in Pakistan included 1,040 participants, 34.4% of whom had good knowledge, 41.7% showed positive attitudes, 57.7% showed good practices, and 69.9% used prevention. Significant findings included the association of gender and education with knowledge (p < 0.05), monthly income with attitudes (p < 0.05), and practices with gender and education (p < 0.05) [20]. Education had a positive impact on KAB in the present population studied in Pakistan. When adjusted for education in the regression analysis, KAB in the 18-30-year age group, better income group, and family with a member in the medical field were positively impacted.

In a cross-sectional survey, Pakistani students’ knowledge was determined to be an average of 76.7%, and only 6.3% had good knowledge. The average attitude score was 68.5%. Knowledge was associated with the type of discipline (p < 0.001), academic degree (p < 0.001), and type of residence of the respondents (p < 0.001). The willingness of the population to be vaccinated was 67.7% [26]. In our population, the vaccine practices for smallpox and COVID-19 were 10.9% (always) and 19.5% (often), respectively. Mpox has received little attention in the context of COVID-19. The Chinese population was shown to be reluctant to adapt to Mpox vaccines [27].

Of the 1,511 respondents, 13.2% reported always watching medical information on TV, and 9.5% had always accessed the Internet to obtain health information. Therefore, these two sources of information are important. Awareness can be created through TV and the Internet. Interest in the United States increased after the WHO declaration of PHEIC for Mpox. People search for most current issues on the Internet. The Internet search for Mpox increased from May 16 to 24, 2022, indicating initial attention to the pandemic within two months of the outbreak. Healthcare workers and policymakers need to provide sources of information that increase awareness among the public and sensitize them [10].

Participants were unsure whether the smallpox vaccine was effective against the Mpox vaccine (14.1%). Perceptions of vaccination and its effectiveness were vague among the public. Similar population behaviors have been reported in previous studies. If vaccines are not compulsory, people may never opt willingly to vaccinate [28]. Participants who had already received the smallpox or COVID-19 vaccines were also likely to have received the Mpox vaccine. The participants had good scores for attitude and practice but poor knowledge scores. The low knowledge scores suggest that there is a need to educate the public about the risks of pandemics and the treatments available for the disease. Attitudes can be modified and practices can be ensured through proper communication. People can easily follow preventive measures if they are fully informed and empowered to make decisions regarding their health decisions. Practices were associated with sex in a previous study (p < 0.05); however, the same positive association was not observed in our study. Family income and preventive attitudes were related in a previous study, and the same association was observed in our study [20].

An important factor identified in our study was the presence of family members who worked in health-related fields. It was positively associated with knowledge of treatment, awareness, and preventive behaviors. The presence of a family member in the medical field has a positive impact on the behavior of people in the house. This increases the knowledge of available treatment options. The presence of a medical professional also enhances behaviors that prevent the spread of the virus. Our study showed a significant association between age, household type, income, and family members in the medical field (p < 0.05). An association with healthcare as a predictor of good knowledge of Mpox has been identified in a previous study. In a study including 480 participants from Saudi Arabia, only 48% of respondents had good knowledge of Mpox. The significant predictors were age, residence, marital status, income, smoking, education, employment status, and association with healthcare (p < 0.01). Participants received information from social media (75%), TV (45%), family and friends (15.6%), and healthcare workers (13.8%). This study highlights the need for intense efforts to promote public awareness and engage them in controlling outbreaks [29].

Marital status had a significant effect on vaccination behavior. Our data showed that married participants were vaccinated less than single people. Binary logistic regression also confirmed the association of certain factors with KAB. When adjusted for gender, education was an insignificant predictor of KAB. Mothers who had an intermediate level of knowledge or less had a negative impact on knowledge. In Pakistan, mothers typically manage their homes. A mother with a good education is more likely to intervene in the preventive practices of the family and play a significant positive role.

Mpox symptoms are mild, and most cases can be treated with supportive care; however, antivirals (e.g., tecovirimat, cidofovir, and brincidofovir) and immunoglobulins (VIGIV) are available for treatment [30]. Immunocompromised patients, children, pregnant women, and breastfeeding mothers may develop complex symptoms. The United States Food and Drug Administration and Health Canada approved tecovirimat for orthopoxviruses. Surveillance data from the 1980s from Africa suggests 85% smallpox vaccine efficacy against Mpox [31-35]. The demographic factors associated with KAB marked as significant in our study are important, and additional predictors need to be identified through in-depth studies with larger data.

Strengths and limitations

The study is a cross-sectional survey about Mpox which is not a pandemic in Pakistan yet but will be a serious public health threat in the future as cases are reported occasionally and several Pakistanis are working in the Middle East or other African countries where Mpox has already revealed its threats. The knowledge of the population is important, and it is imperative to understand and predict the attitude of the population in case there is a pandemic. The study was done on a large scale using every possible source including electronic applications such as WhatsApp to reach the maximum number of people from different areas of the country. The questionnaire was sent as Google Forms which enables easy recording of the responses. The limitation of the study is the language of the questionnaire as we could only include participants who were able to understand English. The population that lacked Internet facilities could not be reached. These limitations impact the generalizability of the study results to a wider population.

## Conclusions

In this study, participants had a good level of knowledge regarding Mpox, especially about the disease, prevention, and testing, although awareness about vaccination locations was lacking. Attitudes toward the risk and severity of Mpox were generally poor, with limited recognition of its severity and low support for preventive measures such as lockdowns. Participants’ behavior was also poor, with low adherence to recommended preventive actions such as quarantine, following restrictions, and receiving vaccinations. These findings highlight the need for targeted public health campaigns to improve both awareness and compliance with preventive measures. The demographic factors associated with KAB marked as significant in our study are important, and additional predictors need to be identified through in-depth studies with larger data. The world has become a global village and knowledge is accessible to most of the population. Therefore, their attitudes can be modified. However, people are unaware of appropriate practices. People obtain false information from the Internet. One Facebook controversy states that images shared by the mainstream media are either edited or from previous African cases. People on Twitter share misinformation about the spread of the virus from powerful people who want to benefit from pharmaceutical companies. Surveys must be conducted to review social media trends, rumors, and sources. The gap between knowledge and practice can be filled by designing strategies that allow the public to access proper information, enabling them to act properly to protect their health during pandemics, such as Mpox. Governments, community leaders, and the social and mainstream media may collaborate to generate multifaceted efforts to improve public knowledge. Therefore, interventions and policies must be designed to address this issue. Populations are important stakeholders in the implementation of public health policies, emphasizing the need for public awareness programs.
